# A case of a young man with secondary hypertension

**DOI:** 10.5339/qmj.2024.53

**Published:** 2024-12-24

**Authors:** Rishabh Baskara Salian, Nidhi Raj, Basavaprabhu Achappa, Suraj Pai, Arfath Ahmed, Svetanshu Sajwan

**Affiliations:** 1 Department of Medicine, Kasturba Medical College, Mangalore, India; 2 Manipal Academy of Higher Education, Karnataka, Manipal, India; 3 Department of Radiodiagnosis and Imaging, Kasturba Medical College, Mangalore, India *Email: raj.nidhi22@gmail.com; 4 Consultant Physician, Kasturba Medical College Hospital, Ambedkar Circle, Mangalore, India; 5 Department of Cardiothoracic and Vascular Surgery, Kasturba Medical College, Mangalore, India

**Keywords:** Hypertension, aorta, renal, coarctation

## Abstract

**Background:**

Secondary hypertension is characterized by an elevated blood pressure greater than 140/90 mmHg, which occurs as a consequence of other diseases. The common etiologies of secondary hypertension include renal parenchymal causes, endocrine disorders, and vascular pathologies like coarctation of the aorta (CoA).

**Case presentation:**

A 20-year-old patient was admitted to our hospital as he complained of headache and palpitations since one week. On examination, the blood pressure in his right upper limb was 180/100 mmHg. The volume of the femoral and the dorsalis pedis pulses was found to be reduced bilaterally. The patient was started on antihypertensive medication labetalol 10 mg injection intravenously immediately. After clinical suspicion and a series of investigations, the patient was diagnosed with severe CoA, distal to the origin of the left subclavian artery via computed tomography (CT) aortogram. The patient was managed by coarctoplasty with stenting.

**Discussion:**

The most striking examination findings indicative of CoA include decreased lower limb pulses and a blood pressure difference of >20 mmHg across both the lower and upper extremities. It is important to evaluate the blood pressure in both upper and lower limbs to diagnose obstructive vascular diseases.

**Conclusion:**

The presence of multiple well-developed collaterals can often mask symptoms and delay the detection of hypertension in patients with CoA. Patients with CoA require regular follow-up to monitor left ventricular outflow tract obstruction, and patients with severe CoA should be treated interventionally to prevent complications including aortic aneurysm and dissection.

The patient was managed by coarctoplasty with stenting and recovered well post-surgery.

## 1. Background

Secondary hypertension is defined as elevated blood pressure greater than 140/90 mmHg, which occurs as a consequence of other diseases. Out of all the patients with hypertension, 10% are found to have a secondary origin.^1^ Identification of the etiology of secondary hypertension is necessary for deciding the treatment. The common etiologies of secondary hypertension include renal parenchymal causes, endocrinal causes, and coarctation of the aorta (CoA) [2].^2^ CoA accounts for approximately 5%–7% of all congenital cardiac diseases, with an estimated incidence of 3 cases per 10,000 births.^3,4^

Embryologically, six pairs of pharyngeal arch arteries form the great vessel system, which includes the aortic arch. These arches appear separately, regress, and eventually transform into components of the great vessel system. The definitive adult aortic arch is the fourth arch, developmental anomalies of which lead to CoA and interrupted aortic arch.^5,6^

CoA is characterized by an anatomical narrowing of the lumen of the aorta. CoA is presently divided into two types pre-ductal and post-ductal depending on the location of the coarctation. Pre-ductal CoA occurs proximal to the ductus arteriosus, whereas post-ductal CoA occurs distal to the ductus arteriosus. Infants and young children are most likely to experience pre-ductal CoA, although post-ductal CoA is uncommon in kids under the age of five.^7^

Upper extremity hypertension is triggered by the narrowing of the aorta, which elevates the blood pressure in the upper limbs. Untreated coarctation can cause early coronary artery disease, aortic aneurysm, or ventricular failure.^8–10^ The presentation of CoA can occur at any age. After the ductus arteriosus has closed, newborns with ductal-dependent circulation can present with cardiac failure, acidosis, and shock. Death can occur quickly in the absence of surgical treatment.^11,12^ Additionally, about 30% of children with CoA still remain undiagnosed upon discharge post-delivery.^13^

Karyotype screening is advised for females who have been diagnosed with CoA, due to its common association with turner syndrome (XO).^14^ CoA has also been linked to a bicuspid aortic valve.^15^ The risk of coarctation and other heart lesions is 10 times higher in children and other first-degree relatives who have been detected to have an obstructive left-sided cardiac lesion.^14,15^ Individuals with a lesser degree of coarctation may not be detected until later in childhood, when a murmur becomes apparent or hypertension is detected. Collateral vessels form out of the internal thoracic arteries, subclavian arteries, and thyrocervical trunks in these individuals and supply blood to the rest of the body.^16,17^

Written informed consent was obtained from the patient for publishing the case report including the pictures of investigation reports.

## 2. Case Presentation

The patient was a 20-year-old man who was admitted to our hospital in March 2023 with chief complaints of headache and palpitations since one week. The headache began two years ago, was intermittent in nature, and aggravated on exertion. The headache increased in the past week with palpitations that were intermittent in nature and were associated with sweating. The patient gave a history of epistaxis two years back which was relieved with medications.

The patient did not complain of fever, neck stiffness, or rigidity. He had no history of previous syncopal attacks and no loss of consciousness. The patient is a nonsmoker and does not consume alcohol, tobacco products, and does not use recreational drugs. No signs of pallor, icterus, cyanosis, clubbing, lymphadenopathy, or edema were seen.

On examination, the right and left radial pulses were 70 beats per minute, regular. The dorsalis pedis pulse and femoral artery pulse were found to be feeble bilaterally. The rate was not recordable. The blood pressure measured in the supine position was 180/100 mmHg in the left arm and 170/100 mmHg was in the right arm, the blood pressure in the left lower limb was not recordable. Pulsations were palpated in the left infrascapular area with the patient bending forward with arms hanging indicating a positive Suzmans sign.^18^

A normal vesicular breath sounds were heard bilaterally. Jugular venous pulsations were normal. S1 and S2 were heard, a systolic murmur was heard on auscultation, the abdomen was soft and non-tender, and there were no focal neurological deficits. A provisional diagnosis of accelerated hypertension was made suspecting renal artery stenosis or CoA or pheochromocytoma. The patient was immediately started on antihypertensive medication Labetalol 10 mg injection intravenously and shifted to the wards with regular monitoring of blood pressure.

The laboratory investigations revealed a normal complete blood count, urine protein/24 hours was elevated, and the rest of the urine analysis was normal. Thyroid function test and liver function test levels were normal, arterial blood gas analysis was normal on initial presentation (Table 1).

## 3. Investigative Procedure

An electrocardiogram revealed sinus tachycardia (heart rate 114 beats per minute), left axis deviation indicating left ventricular hypertrophy. 2D echocardiography revealed mild mitral regurgitation, concentric left ventricular hypertrophy, ejection fraction of 60%, no CoA, and no patent ductus arteriosus.

**Figure 1.**
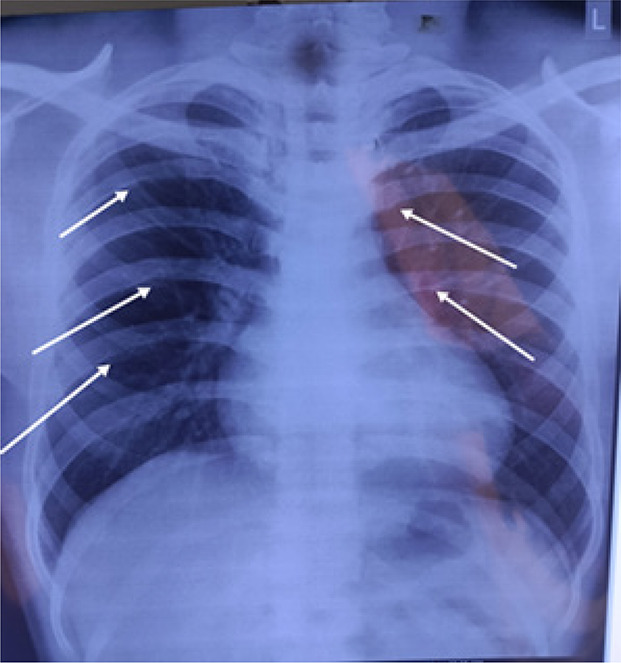
Plain Xray Posteroanterior view of the chest showing enlarged heart shadow notching of the ribs.

A chest X-ray was also done as a routine investigation which showed enlarged heart shadow and notching of the ribs (Figure 1).

**Figure 2.**
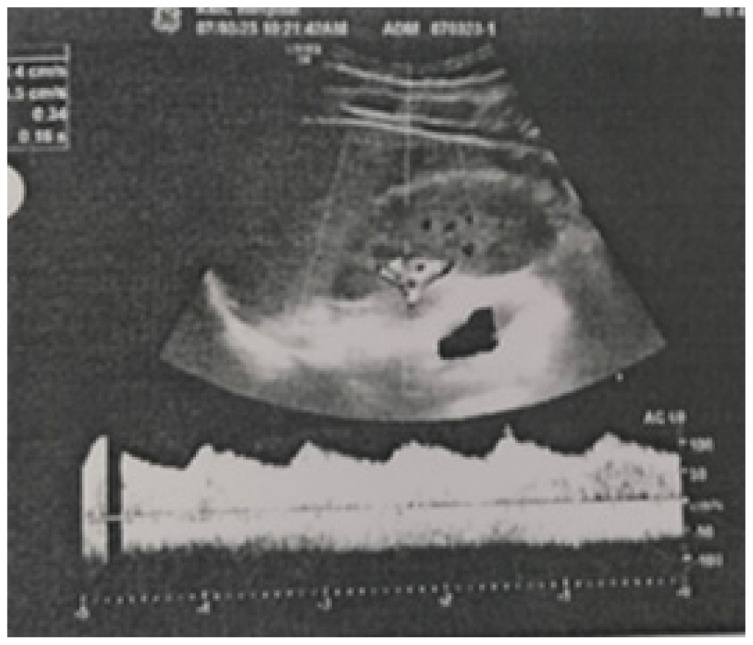
Doppler ultrasound showing Tardus Parvus waveforms.

A doppler study of the bilateral renal arteries showed Tardus Parvus waveforms in the bilateral intra-renal arterial system indicating reduced magnitude of blood flow (Figure 2).

**Figure 3.**
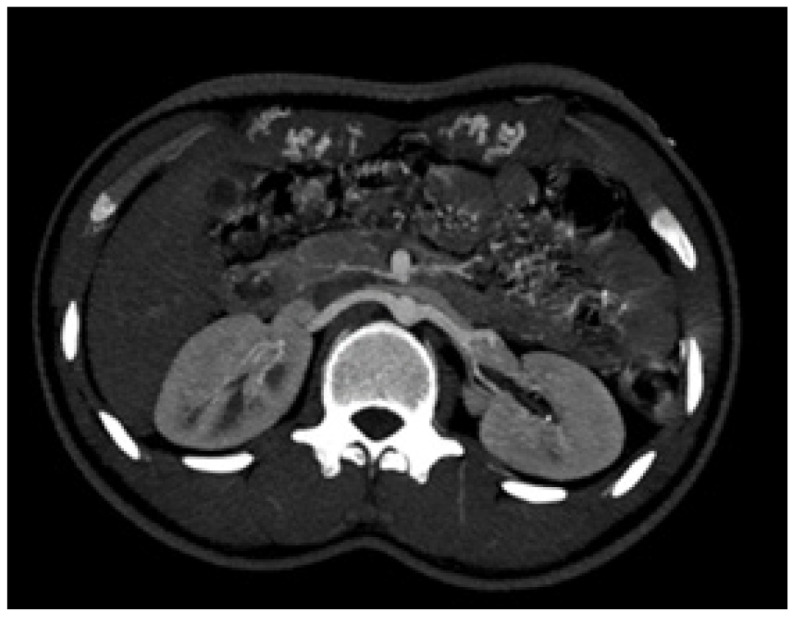
Computed tomography renal axial section showing normal renal arteries.

**Figure 4.**
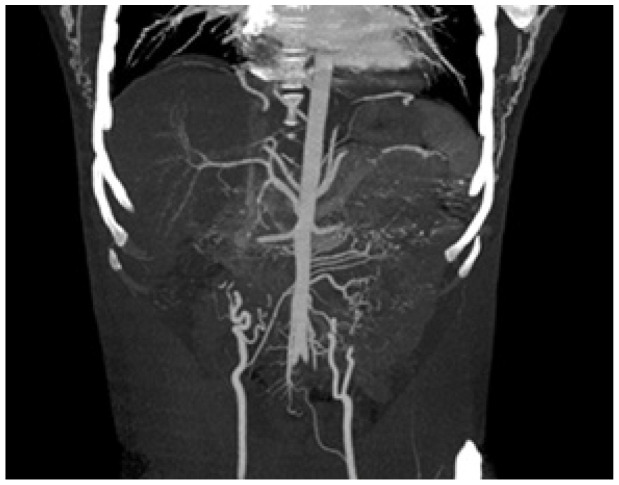
Computed tomography renal coronal section showing collaterals.

A nephrology referral was sought in view of the renal artery doppler changes and to evaluate for aldosterone axis. CT renal angiogram revealed normal sized kidneys (Figure 3). Prominent bilateral internal mammary arteries with multiple branches anastomosing with branches arising from dilated inferior epigastric arteries in anterior abdominal wall were noted (Figure 4).

**Figure 5.**
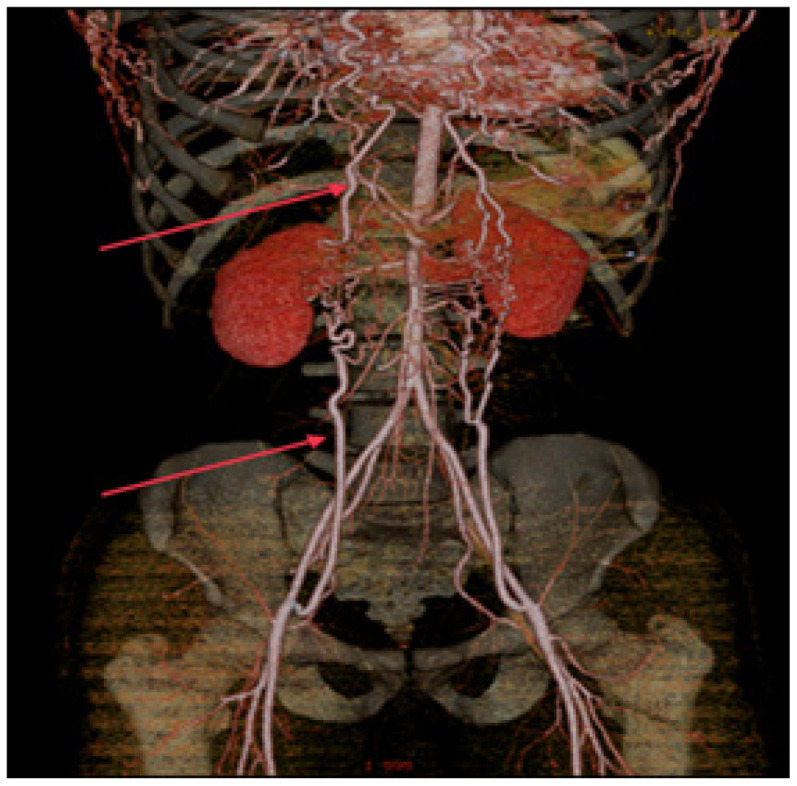
Volume rendering technique showing anastomoses between internal mammary arteries and inferior epigastric arteries.

The volume rendering technique showed anastomoses between the internal mammary arteries and the inferior epigastric arteries (Figure 5).

**Figure 6.**
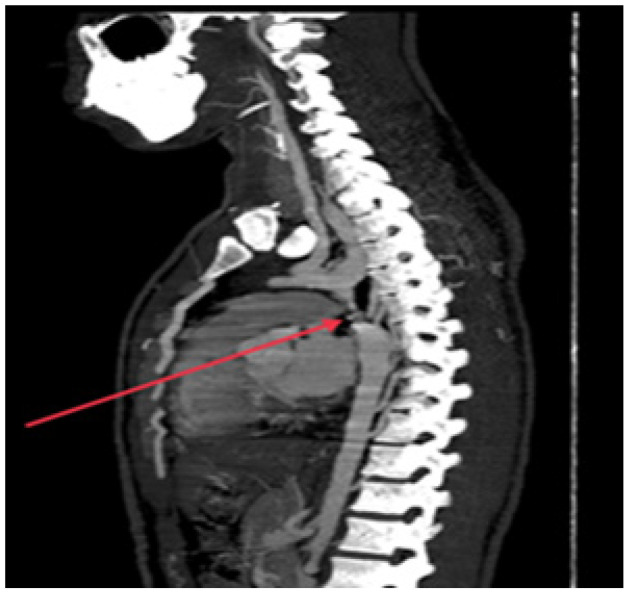
Computed tomography thorax sagittal section showing coarctation of the aorta.

A computed thoracic aortogram revealed a diffuse smooth narrowing of the arch of the aorta distal to the origin of the left subclavian artery. The minimum luminal diameter was 2.9 mm and the length of the narrowing was 1.7 cm from the origin of the left subclavian artery. Reformation of the descending thoracic aorta distal to the narrowing and multiple collateral circulations was seen, which suggest severe CoA (Figure 6).

An ophthalmology referral was sought to rule out hypertensive retinopathy. Dilated fundus examination revealed arterial attenuation, and crossing changes were seen bilaterally. Macula was normal. The patient was diagnosed with bilateral Grade II hypertensive retinopathy.

The patient was further managed by coarctoplasty with stenting, and he was discharged seven days post-operation. The patient was advised to return to the hospital for regular follow-up visits.

## 4. Discussion

Hypertension is one of the most characteristic presenting symptoms for people with CoA who go into adulthood undiagnosed.^17^ Patients may express regular headache complaints or claudication in the lower limbs after exertion. The most striking examination findings indicative of coarctation in these individuals include decreased and/or delayed lower limb pulses and a blood pressure difference >20 mmHg across both the lower and upper extremities.^17^ However, the tibial and femoral pulses may only be slightly reduced in patients with significant collateral blood flow.^19^

It is necessary to evaluate all patients for the cause of hypertension, but since it is not cost-effective to investigate every patient for the origin of hypertension, there are several factors that indicate the possibility of secondary hypertension.^20^ According to the 2018 guidelines for the management of arterial hypertension by the European Society of Cardiology and European Society of Hypertension,^21^ screening for the cause of hypertension should be considered in (i) patients <40 years of age diagnosed with grade 2 hypertension or childhood onset of hypertension, (ii) resistant hypertension - blood pressure that remains >140/90 mmHg after treatment with the optimal doses of three or more drugs, which includes an angiotensin-converting enzyme inhibitor (ACEI) inhibitor or an angiotensin receptor blocker (ARB) with a thiazide and a calcium channel blocker,^20,21^ (iii) biochemical or clinical features indicating endocrinal causes of hypertension, (iv) symptoms of pheochromocytoma, and (v) clinical features suggestive of obstructive sleep apnea.

Early detection of secondary hypertension and intervention is crucial to prevent complications such as cerebral hemorrhage, aortic dissection, endocarditis, or concomitant aortic valve disease.

In this patient, although coarctation was highly suspected, the echocardiography did not reveal coarctation. The missed diagnosis by echocardiography was subsequently followed by a contrast-enhanced CT aortogram clearly showing severe coarctation in the descending aorta, distal to the origin of the subclavian artery, which resulted in the final diagnosis. The patient underwent the surgical treatment via coarctoplasty with stenting. The surgical options for treatment for CoA include resection with end-to-end anastomosis, bypass graft insertion, subclavian flap aortoplasty, and endovascular repair.^22^

## 5. Conclusion

The presence of multiple well-developed collaterals can often mask symptoms and delay the detection of hypertension in patients with CoA. These collateral vessels serve as alternative pathways for blood flow, compensating for the obstruction in the aorta and maintaining adequate perfusion to vital organs. As a result, patients may remain asymptomatic or experience only mild symptoms for an extended period, as seen in this case. It highlights the importance of considering CoA as a potential underlying cause of hypertension, especially in young adults with atypical or minimal symptoms.

Patients with CoA require regular follow-up in order to monitor left ventricular outflow tract obstruction and progression of complications of hypertension and improve long-term outcomes. The patient was managed by coarctoplasty with stenting and recovered well post-surgery.

## Conflict of Interest Statement

The authors declare that they have no competing interests.

## Author Contributions

RBS, NR, and BA were the main contributors to the concept and design of the report, and they critically revised the final draft of the article. SS, AA, and SP collected the case report data from the patient records and drafted the article. All authors critically revised and approved the final manuscript for publication.

## Consent for Publication

An informed consent was obtained from the patient for publication of the case.

## Figures and Tables

**Table 1.** Patient’s blood and urine analysis reports.

**Test name**	**Results**	**Normal values**
	Complete blood count	
Haemoglobin	14.4	13.0–17.0 g/dL
Red blood cell count	4.96	4.50–5.50 million/cum
White blood cell count	8000	4000–10000 cells/cum
Platelet count	321000	150000–400000
	Liver function tests	
Total bilirubin (serum)	0.45	0.30–1.20 mg/dL
Albumin (serum)	4.6	3.50–5.20 g/dL
Globulins (serum)	2.51	1.80–3.40 g/dL
Aspartate aminotransferase (serum)	23	0.00–40.00 U/L
Alanine transaminase (serum)	25	0.00–41.00 U/L
Alkaline phosphatase (ALP)	102	35.00–130.00 U/L
	Urine analysis	
Urine volume (24 hours)	1800	mL
Urine protein	16.4	14.00–138.00 mg/dL
Urine specific gravity	1.01	1.003–1.035
	Thyroid function tests	
Serum free T4	1.24	0.930–1.710 ng/dL
Serum free T3	5.12	2.000–4.400 pg/mL
	Arterial blood gas analysis	
Serum sodium	138	136.0–149.0 mmol/L
Serum potassium	3.81	3.50–5.30 mmol/L
Serum bicarbonate	25.6	23.0–27.0 mmol/L
	Renal function tests	
Serum creatinine (serum)	0.74	0.60–1.30 mg/dL
Uric acid (serum)	3.9	3.5–7.0 mg/dL
Blood urea (serum)	17	16.6–48.0 mg/dL
